# Natural history of the ERVWE1 endogenous retroviral locus

**DOI:** 10.1186/1742-4690-2-57

**Published:** 2005-09-22

**Authors:** Bertrand Bonnaud, Jean Beliaeff, Olivier Bouton, Guy Oriol, Laurent Duret, François Mallet

**Affiliations:** 1 UMR 2714 CNRS-bioMérieux, IFR128 BioSciences Lyon-Gerland Ecole Normale Supérieure de Lyon, 46 allée d'Italie, 69364 Lyon cedex 07, France; 2 Laboratoire de Biométrie et Biologie Evolutive, UMR CNRS 5558, Université Claude Bernard – Lyon 1, 43 Bd du 11 Novembre 1918, 69622 Villeurbanne Cedex, France

## Abstract

**Background:**

The human HERV-W multicopy family includes a unique proviral locus, termed ERVWE1, whose full-length envelope ORF was preserved through evolution by the action of a selective pressure. The encoded Env protein (Syncytin) is involved in hominoid placental physiology.

**Results:**

In order to infer the natural history of this domestication process, a comparative genomic analysis of the human 7q21.2 syntenic regions in eutherians was performed. In primates, this region was progressively colonized by LTR-elements, leading to two different evolutionary pathways in Cercopithecidae and Hominidae, a genetic drift *versus *a domestication, respectively.

**Conclusion:**

The preservation in Hominoids of a genomic structure consisting in the juxtaposition of a retrotransposon-derived MaLR LTR and the ERVWE1 provirus suggests a functional link between both elements.

## Background

The infectious retrovirus founding the contemporary HERV-W family [1] entered the genome of a Catarrhine ancestor 25–40 million years ago [2,3]. The spread of the HERV-W family into the genome essentially results from autonomous and non-autonomous events of intracellular retrotransposition of transcriptionally active copies [4,5]. The HERV-W family contains a unique locus, termed ERVWE1, which encodes an envelope glycoprotein expressed in the placenta [3,6]. This envelope, also dubbed Syncytin, exhibits fusogenic properties *in vitro *and is directly involved in trophoblast differentiation [6-8]. The functional conservation of the ERVWE1 locus among Hominoids [9] and the identification of selective constraints on the *env *gene [10] strongly suggest that this retroviral locus has been recruited to play a role in placental physiology. In order to decipher the natural history of the ERVWE1 locus, we performed a comparative genomic analysis of the eutherian chromosomal regions syntenic to a portion of human chromosome 7q21.2 containing the (H)ERVWE1 locus. We observe in this region that the content in transposable elements varies between species, notably with a progressive enrichment of LTR-elements in the Platyrrhine and Catarrhine lineages. Based on an ancestral mosaic of LTR-elements, this retroviral cluster followed two opposed evolutionary pathways, a genetic drift *versus *a domestication, in Cercopithecidae and Hominidae lineages, respectively.

## Results and Discussion

**Figure 1:**
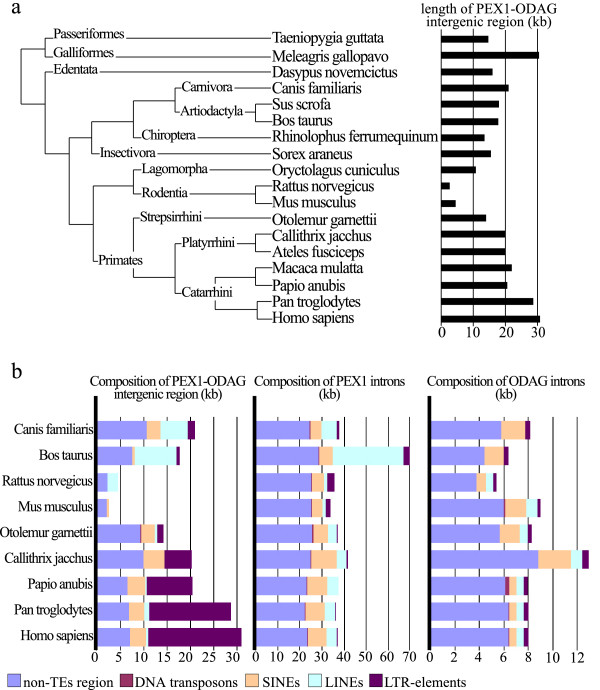
**Comparative analysis of PEX1-ODAG orthologous locus. **(a) Length of PEX1-ODAG intergenic region. Species with an identified PEX1-ODAG intergenic region (either extracted from databases or sequenced in the lab) are indicated on the tree. Clades are redrawn from a previous mammalian phylogeny [23]. Branches are not drawn to scale. The length of the PEX1-ODAG intergenic region is indicated for each species. (b) Length and TEs composition of PEX1 and ODAG intergenic and intronic regions. Species were selected regarding the quality of TEs description in RepBase) [18].

The initial failure to isolate the ERVWE1 integration site in Old World Monkeys [9] suggested that this region was shaped by complex recombination events. The comparative analysis of human ERVWE1 flanking sequences with the mouse genome has revealed two syntenic anchor points in the ERVWE1 provirus vicinity. Thus, the peroxisome biogenesis factor 1 gene (PEX1) and the ocular development-associated gene (ODAG) are located upstream and downstream from ERVWE1, respectively. In genomic databases, the genetic linkage between both boundary genes was found in 14 mammals and 2 birds (Figure 1a). In addition, to fill in the evolutionary gap of this dataset, we PCR amplified and sequenced the intergenic region of two primates, Macaca mulatta and Ateles fusciceps robustus.

The length of the PEX1-ODAG intergenic region varies among species (17.8 ± 7.9 kb), ranging from 2.6 kb to 30.9 kb for rat and human, respectively (Figure 1a). The length variation of the intergenic region is generally due to the presence of various transposable elements (TEs) (Figure 1b). The particularly short intergenic regions of rodents may result from the general deletion mechanisms previously proposed to account for rodent small genome size [11]. The herein described region suggests that the rodent deletion process show no bias towards TEs (Figure 1b). In comparison, the length of PEX1 and ODAG intronic regions is homogenous (PEX1 : 38.5 ± 13.4 kb ; ODAG : 8.1 ± 2.5 kb), the variability relying mostly upon one species for each gene (Figure 1b). For example, the largest intronic region of PEX1 orthologous gene is observed in Bos taurus and corresponds to the presence of about 40 kb of TEs as compared to 10–20 kb in other species (Figure 1b).

TEs contents differ quantitatively and qualitatively between lineages and between intergenic and intronic regions (Figure 1b). In introns, SINEs then LINEs represent the majority of TEs among all species. The singular large LINE content of Bos taurus PEX1 introns is compatible with the huge amount of specific LINE elements in the genome of this species [12]. The absence of such specific LINE elements in Bos taurus ODAG introns may be due to the shorter length of this gene. Within the intergenic regions, first LINEs and second SINEs predominate in Carnivores, Artiodactyls and Rodents. In primates, the intergenic regions consist largely of LTR elements and Alus. The LTR-elements are clustered in a 20 kb region just downstream from the PEX1 gene and the Alu elements are spread within the 10 kb region upstream from the ODAG gene. This local LTR concentration in primates is particularly high as compared to previous comparative analysis over several megabases [12]. The 30 kb human PEX1-ODAG intergenic region contains 11%, 2% and 64% of Alus, LINE-1s and LTR-elements, respectively.

**Figure 2:**
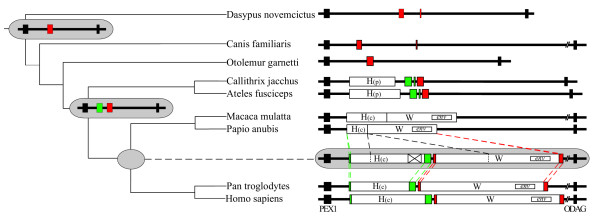
**Phylogenetic analysis of the PEX1-ODAG intergenic region in 9 mammal species. **Flanking black boxes correspond to the 24^th ^exon and the 5^th ^exon of the PEX1 and ODAG genes, respectively. LTR elements are depicted as red boxes (MaLR-e1 LTR), green boxes (ERV-P LTR) and empty boxes (ERVWE1 and ERV-H proviruses). The ERVWE1 provirus is labeled W, ERV-H Platyrrhini and Catarrhini lineage specific proviruses are labeled H(p) and H(c), respectively. *env *smaller boxes refer to the ERVWE1 *env *gene. Proposed ancestral chromosomal structure are drawn in grey cartouches. The cross-box within the H(c) ancestor represents a pol/env deletion as referenced to the HERV-H repbase consensus. Dash lines represent the evolutionary processes leading to Cercopitheque vs. Hominoid lineages. The double slashes indicate the truncation of longest sequences. Clades are derived from previous phylogeny [23] and branches are not drawn to scale.

The picture obtained from the comparison of the syntenic PEX1-ODAG intergenic regions between mammalian species is informative about the putative composition of this region in common ancestors, depicted at the nodes of the phylogenic tree (Figure 2). In addition, LTR-element flanking sequences indicate whether the retrotransposition process was autonomous, i.e. mediated by an HERV-specific reverse transcriptase (RT), or non-autonomous, i.e. mediated by the LINE RT which contributes to pseudogene formation. The autonomous events leads to the duplication of a genomic 4–6 bp sequence, flanking consequently the proviral 5' and 3' LTRs. In the case of LINE RT retrotransposition, a longer flanking repeat of 10–16 bp is observed together with an mRNA typical structure (absence of promoter element and presence of a 3' poly(A) tail) [13,14]. By merging all this information, we infer the natural history of this region.

**Figure 3:**
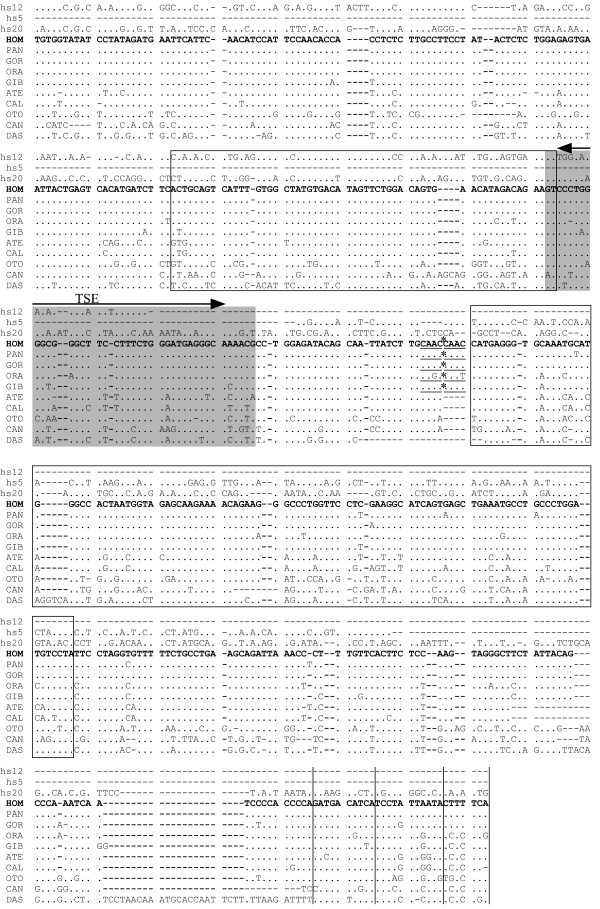
**Alignments of orthologous and paralogous MaLR-e1 LTR sequences from mammalian species. **Sequences were assembled using the human sequence (HOM) as reference. Orthologous sequences are from the following origin: HOM: Homo sapiens, PAN: Pan troglodytes, GOR: Gorilla gorilla, ORA:Pongo pygmaeus, GIB: Hylobatides pileatus, ATE: Ateles fusciceps robustus, CAL: Callithrix jacchus, OTO: Otolemur garnetti, CAN: Canis familiaris, DAS: Dasypus novemcictus. Hs12, hs5 and hs20 correspond to MaLR-e1 putative paralogous sequences isolated in the human genome. 5' and 3' openboxes corresponds to MLT1J2 and MLT1J Repbase consensuses, respectively. The region with grey background indicates the 5' or 3' boundaries zone of most partial MaLR-e1 in human and dog genomes. Four putative 3' boundaries of the MaLR-e1 LTR are shown as vertical bars. The double head arrow delimits the trophoblastic specific enhancer (TSE). * correspond to the location of the omitted ERVWE1 provirus. Direct repeats flanking the ERVWE1 integration site are underlined. Bold gray characters in the 3' end of DAS and CAN sequences precede large insertions (1,3 kb and 4,5 kb, respectively) omitted in the alignment.

The first step of the parsimonious scenario consists in the integration of mammalian apparent LTR-retrotransposon (MaLR) element in the PEX1-ODAG intergenic region of a primitive mammalian ancestor, followed by a local recombination between the 5' and 3' paired LTRs)[15], generating the MaLR isolated LTR. However, the absence among species of flanking duplicated sequences as a vestige of the original integration does not support this hypothesis, although this 100 million years-old signature may have vanished. In human, only two short 57 bp and 106 bp segments were identified (Figure 3), presenting 75.4 % and 67.9% similarity with MLT1J2 and MLT1J subfamilies of MaLR elements)[15], respectively. The 260 bp remaining parts of the MaLR LTR exhibits no similarity with previously defined MaLR consensus sequences, suggesting the identification of a new MaLR subfamily named MaLR-e1. In addition, similarity search (threshold 60%) of MaLR-e1 human and dog sequences on their respective genomes indicate only one other full-length element and a vast majority of elements consisting roughly in either the 5' or the 3' half part of MaLR-e1. The location of one end of these MaLR partial sequences within a 40 bp region (Figure 3) bordered on each side by the MLT1J and MLT1J2 identified regions suggests an authentic chimerical origin for this MaLR-e1 LTR. The paucity of the MaLRs bipartition reflect an unsuccessful propagation of this form. Strikingly, the deduced junction area of both parts of the chimera corresponds to a functional sequence consisting of a trophoblast specific enhancer (TSE) [16].

Second, a 633 bp ERV-P element was acquired by the common ancestor of the Platyrrhines and Catarrhines more than 40 million years ago [17]. As for the MaLR-e1 element, the absence of trivial duplication of the integration site shades the origin of the contemporary isolated ERV-P LTRs. In any case, the putative primary recombination between paired LTRs may have occurred rapidly after integration as no ERV-P internal sequence can be detected in any of the studied species. The LTR sequence is complete as referred to the consensus sequence)[18], although the 5' first ten nucleotides largely diverged.

Third, ERV-H and ERV-W proviruses integrated in the germ line of a Catarrhine ancestor, within the ERV-P and MaLR-e1 LTRs, respectively. Note that an ERV-H sequence is identified in the Platyrrhines (ERV-H(p)), distinct from the Catarrhines ERV-H provirus (ERV-H(c)) described above, as located about 2 kb upstream from the ERV-P LTR. The ERV-W element corresponds to the ERVWE1 provirus as it contains the locus-specific signature (a 12 bp deletion in the 3' end of the *env *gene) previously identified by comparing (H)ERVWE1 and paralogous HERV-W copies [10]. The presence in several species of degenerated direct repeat at both ends of ERV-H(c) [A(C/T)(G/A)AC] and ERVWE1 [CA(A/G)(C/T)] proviruses attests that retrovirus-like integration events occurred. Whether these proviral insertions derived from re-infection or cis- or trans-retrotransposition processes remains unknown. Nevertheless, the duplication of the integration site indicates the existence at that time of functional H- and W-specific reverse transcriptases. The accumulation of independent substitutions in 5' and 3' paired LTRs, identical when the provirus integrated, is informative about the chronology of these events. Thus, the comparison of paired LTRs distances between the ERV-H(c) and the ERVWE1 proviruses (0.84 and 0.65, respectively) suggests that ERV-H(c) integrated earlier than ERVWE1.

Then the Catarrhine ancestor genomic structure followed two divergent evolutionary pathways in Cercopitheques and Hominoids (Figure 2). An about 9 kb fragment was deleted in the Cercopitheque lineage, consisting of a 3.8 kb *pol-env*-LTR ERV-H(c) sequence, a 4.3 kb LTR-*gag-pol *ERVWE1 sequence and the 0.9 kb inter-proviral region. This large deletion produced an hybrid ERV-(H/W) defective proviral structure. Surprisingly, as both ERV-H(c) 5' and ERVWE1 3' flanking sequences were also deleted, the Cercopitheque lineage is devoid of MaLR-e1 and ERV-P LTRs elements. This global inactivation of all four LTR elements was followed by the genetic drift of the *env *gene as revealed by the presence of different inactivating substitutions in the baboon and macaque ERVWE1 remnants, a stop codon in position 181 and a frameshift in position 498, respectively. In Hominoids, the overall 30 kb structure was preserved as confirmed by overlapping LD-PCR amplification of gorilla, orangutan and gibbon genomic DNA (data not shown). In Hominoids, the ERV-H(c) element contains a locus specific signature that consists in a unique *pol*-*env *junction. An accurate dating of this deletion event would require an extended panel of species as the region of interest is absent from the Macaca mulatta and Papio anubis genomes. The presence of the *env *12 bp deletion (crucial for the Env fusogenic activity) in Hominoids [10] and Cercopitheques ERVWE1 proviruses suggests that this deletion occurred originally in a primary Catarrhine ancestor possibly soon after integration, in the youth of the ERV-W family. Furthermore, the ERVWE1 *env *signature was found to be unique in human and chimpanzee genomes, what shows an absence of retrotransposition of this element. This suggests an absence of expression of the ERVWE1 locus in the Hominoid germ line, as opposed to many other HERV-W loci that were shown to retrotransposed using mainly LINE-RT [5].

ERVWE1 was shown to be a bona fide gene involved in hominoid placental physiology [9]. The concomitant conservation in Hominoids of the surrounding LTR elements suggests that they were either required for ERVWE1 activity or hitchhiked during the purifying ERVWE1 selection process [10]. The substitution profile along the whole region does not rule out any hypotheses. Nevertheless, it reveals the strict identity of the MaLR-e1 portion located upstream from ERVWE1 in human, chimpanzee and gorilla, as opposed to a MaLR-e1 3' part different for each species. The regulation of the expression of ERVWE1 *env *was shown to be a bipartite element [16] composed of (i) a cyclic AMP (cAMP)-inducible retroviral promoter, the ERVWE1 5' LTR, and (ii) a 436 bp upstream regulatory element (URE), encompassing the MaLR-e1 5' part, that contains the trophoblast specific enhancer (TSE) cited above, conferring high level of expression and placental tropism [16]. Although efficient, the cooperation between the URE and the LTR seemed complex due to an interference phenomenon, probably resulting from the presence of AP-2 and Sp-1 binding sites on the TSE and the cAMP-responsive elements of the LTR [16]. Interestingly, the gibbon transcriptional regulatory elements shows an *in vitro *biased behavior as compared to human, chimpanzee, gorilla and orangutan orthologous elements, i.e. the ERVWE1 5' LTR exhibits a higher placental promoter activity [9] and the URE is deficient in enhancer activity [16]. This feature of the gibbon URE seems associated with two specific mutations in AP-2 and Sp-1, an enhancer activity equivalent to the human one being restored after the modification of the two corresponding residues [16]. Although we cannot exclude the possibility that these observations are partially due to the specific context of a human trophoblastic cell line, this functional analysis supports the very recent recruitment of the elderly MaLR-e1 5' half as proposed in this work. Thus, a LTR of retrotransposon MaLR element and a LTR of a (H)ERV-W proviral locus were co-opted to regulate *syncytin *expression in placenta. Interestingly, the newly identified murine syncytin-B *env *gene which triggers cell-cell fusion *in vitro *and is expressed specifically in placenta *in vivo *displays an upstream MaLR LTR [19]. Whether this represents an additional element to the puzzling convergent physiological role of primate and rodent syncytins remains to be determined.

## Conclusion

We observe in the region syntenic to a portion of human chromosome 7q21.2 containing the (H)ERVWE1 locus a progressive enrichment of LTR-elements in the Platyrrhine and Catarrhine lineages. Based on an ancestral mosaic of LTR-elements, two opposed evolutionary pathways are followed, a genetic drift *versus *a domestication, in Cercopithecidae and Hominidae lineages, respectively. The domestication process includes the ERVWE1 locus in Hominoid species, and putatively a retrotransposon-derived MaLR LTR strictly conserved in the Homo/Pan/Gorilla subgroup. We propose that both elements were recruited to achieve the regulation of *syncytin *expression in placenta.

## Methods

Syntenic sequences to PEX1-ODAG intergenic regions are extracted from the high throughput genomic sequences (HTGS) division of GenBank using BLAST [20]. The query sequence is composed of exons of PEX1 and ODAG genes, as described in the ensembl repository  as vega transcript OTTHUMT00000060247 and OTTHUMG00000023913, respectively. We obtain the following GenBank accession nos., [GenBank:AC092510.2]: Papio anubis, [GenBank:AC148267.2] and [GenBank:AC148269.3]: Callithrix jacchus, [GenBank:AC148127.3] and [GenBank:AC149006.1]: Otolemur garnettii, [GenBank:AC147739.3]: Dasypus novemcinctus, [GenBank:AC148524.3]: Rhinolophus ferrumequinum, [GenBank:AC145009.2] and [GenBank:AC108896.2]: Bos taurus, [GenBank:AC105371.2]: Sus scrofa, [GenBank:AC147729.2]: Oryctolagus cuniculus, [GenBank:AC148352.2]: Sorex araneus, [GenBank:AC097829.7], [GenBank:AC079989.2], [GenBank:AC127809.3] and [GenBank:AC079998.2]: Rattus norvegicus, [GenBank:AC092872.2]: Pan troglodytes, [GenBank:AC114335.3]: Canis familiaris, [GenBank:AC148249.3]: Otolemur garnettii, [GenBank:AC148380.2] and [GenBank:AC148379.2]: Taeniopygia guttata, [GenBank:AC148423.3] and [GenBank:AC148421.2]: Meleagris gallopavo, [GenBank:AC138736.2]: Gallus gallus.

We use RepeatMasker (Smit, AFA, Hubley, R & Green, P. *RepeatMasker Open-3.0*. 1996–2004 ) to identify transposable elements in all the studied species. Sequence alignments were computed with ClustalW [21] and refined manually using Seaview [22].

We have sequenced Ateles fusciceps robustus and Macaca mulatta genomic PEX1-ODAG region. Sequences are provided in genomic databases with the following accession number : [GenBank:AY925147] for Ateles fusciceps robustus and [GenBank:AY925148] for Macaca mulatta.

## List of Abbreviations

HERV: human endogenous retrovirus

ORF: open reading frame

LTR: long terminal repeat

MaLR: mammalian apparent LTR-retrotransposon

SINE: short interspersed element

LINE: long interspersed element

LD-PCR: long distance PCR

## Competing interests

The author(s) declare that there are no competing interests.

## Authors' contributions

BB designed this study and edited the manuscript. JB, OB and GO isolated and sequenced Macaca mulatta and Ateles fusciceps robustus PEX1-ODAG regions. They also participated to the sequence analysis. LD and FM conceived of the study, and participated in its design and coordination and helped to draft the manuscript.

## References

[B1] Blond JL, Beseme F, Duret L, Bouton O, Bedin F, Perron H, Mandrand B, Mallet F (1999). Molecular characterization and placental expression of HERV-W, a new human endogenous retrovirus family. J Virol.

[B2] Kim HS, Takenaka O, Crow TJ (1999). Isolation and phylogeny of endogenous retrovirus sequences belonging to the HERV-W family in primates. J Gen Virol.

[B3] Voisset C, Bouton O, Bedin F, Duret L, Mandrand B, Mallet F, Paranhos-Baccala G (2000). Chromosomal distribution and coding capacity of the human endogenous retrovirus HERV-W family. AIDS Res Hum Retroviruses.

[B4] Costas J (2002). Characterization of the intragenomic spread of the human endogenous retrovirus family HERV-W. Mol Biol Evol.

[B5] Pavlicek A, Paces J, Elleder D, Hejnar J (2002). Processed pseudogenes of human endogenous retroviruses generated by LINEs: their integration, stability, and distribution. Genome Res.

[B6] Blond JL, Lavillette D, Cheynet V, Bouton O, Oriol G, Chapel-Fernandes S, Mandrand B, Mallet F, Cosset FL (2000). An envelope glycoprotein of the human endogenous retrovirus HERV-W is expressed in the human placenta and fuses cells expressing the type D mammalian retrovirus receptor. J Virol.

[B7] Mi S, Lee X, Li X, Veldman GM, Finnerty H, Racie L, LaVallie E, Tang XY, Edouard P, Howes S (2000). Syncytin is a captive retroviral envelope protein involved in human placental morphogenesis. Nature.

[B8] Frendo JL, Olivier D, Cheynet V, Blond JL, Bouton O, Vidaud M, Rabreau M, Evain-Brion D, Mallet F (2003). Direct involvement of HERV-W Env glycoprotein in human trophoblast cell fusion and differentiation. Mol Cell Biol.

[B9] Mallet F, Bouton O, Prudhomme S, Cheynet V, Oriol G, Bonnaud B, Lucotte G, Duret L, Mandrand B (2004). The endogenous retroviral locus ERVWE1 is a bona fide gene involved in hominoid placental physiology. Proc Natl Acad Sci U S A.

[B10] Bonnaud B, Bouton O, Oriol G, Cheynet V, Duret L, Mallet F (2004). Evidence of Selection on the Domesticated ERVWE1 env Retroviral Element Involved in Placentation. Mol Biol Evol.

[B11] Waterston RH, Lindblad-Toh K, Birney E, Rogers J, Abril JF, Agarwal P, Agarwala R, Ainscough R, Alexandersson M, An P (2002). Initial sequencing and comparative analysis of the mouse genome. Nature.

[B12] Thomas JW, Touchman JW, Blakesley RW, Bouffard GG, Beckstrom-Sternberg SM, Margulies EH, Blanchette M, Siepel AC, Thomas PJ, McDowell JC (2003). Comparative analyses of multi-species sequences from targeted genomic regions. Nature.

[B13] Wei W, Gilbert N, Ooi SL, Lawler JF, Ostertag EM, Kazazian HH, Boeke JD, Moran JV (2001). Human L1 retrotransposition: cis preference versus trans complementation. Mol Cell Biol.

[B14] Esnault C, Maestre J, Heidmann T (2000). Human LINE retrotransposons generate processed pseudogenes. Nat Genet.

[B15] Smit AF (1993). Identification of a new, abundant superfamily of mammalian LTR-transposons. Nucleic Acids Res.

[B16] Prudhomme S, Oriol G, Mallet F (2004). A retroviral promoter and a cellular enhancer define a bipartite element which controls env ERVWE1 placental expression. J Virol.

[B17] Goodman M, Porter CA, Czelusniak J, Page SL, Schneider H, Shoshani J, Gunnell G, Groves CP (1998). Toward a phylogenetic classification of Primates based on DNA evidence complemented by fossil evidence. Mol Phylogenet Evol.

[B18] Jurka J (2000). Repbase update: a database and an electronic journal of repetitive elements. Trends Genet.

[B19] Dupressoir A, Marceau G, Vernochet C, Benit L, Kanellopoulos C, Sapin V, Heidmann T (2005). Syncytin-A and syncytin-B, two fusogenic placenta-specific murine envelope genes of retroviral origin conserved in Muridae. Proc Natl Acad Sci U S A.

[B20] Altschul SF, Gish W, Miller W, Myers EW, Lipman DJ (1990). Basic local alignment search tool. J Mol Biol.

[B21] Thompson JD, Higgins DG, Gibson TJ (1994). CLUSTAL W: improving the sensitivity of progressive multiple sequence alignment through sequence weighting, position specific gap penalties and weight matrix choice. Nucleic Acids Res.

[B22] Galtier N, Gouy M, Gautier C (1996). SEA VIEW and PHYLO_WIN: two graphic tools for sequence alignment and molecular phylogeny. Comput Appl Biosci.

[B23] Murphy WJ, Eizirik E, O'Brien SJ, Madsen O, Scally M, Douady CJ, Teeling E, Ryder OA, Stanhope MJ, de Jong WW (2001). Resolution of the early placental mammal radiation using Bayesian phylogenetics. Science.

